# Wide-area Epidemics of Influenza and Pediatric Diseases from Infectious Disease Surveillance in Japan, 1999-2005

**DOI:** 10.2188/jea.17.S23

**Published:** 2008-01-30

**Authors:** Yoshitaka Murakami, Shuji Hashimoto, Akiko Ohta, Miyuki Kawado, Michiko Izumida, Yuki Tada, Mika Shigematsu, Yoshinori Yasui, Kiyosu Taniguchi, Masaki Nagai

**Affiliations:** 1 Department of Health Science, Shiga University of Medical Science.; 2 Department of Hygiene, Fujita Health University School of Medicine.; 3 Department of Public Health, Saitama Medical University Faculty of Medicine.; 4 Infectious Disease Surveillance Center, National Institute of Infectious Diseases.

**Keywords:** Communicable Diseases, Sentinel Surveillance, Disease Outbreaks, Influenza, Human

## Abstract

**BACKGROUND:**

Epidemics of infectious diseases usually start in small areas and subsequently become widespread widely. Although a method for detecting epidemics in public health center (PHC) areas has been proposed and used in the National Epidemiological Surveillance of Infectious Diseases in Japan, wide-area epidemics have not been fully investigated.

**METHODS:**

Using the abovementioned method, we defined an epidemic as that occurring for a week in at least one PHC area in a prefecture and a wide-area epidemic as that when the number of people living in epidemic PHC areas exceeds 30% of the prefectural population. The number of weeks of an epidemic or wide-area epidemic for influenza and 11 pediatric diseases was observed in 47 prefectures in Japan from 1999 through 2005.

**RESULTS:**

Epidemics and wide-area epidemics of influenza occurred for an average of 7.0 and 4.3 weeks in a year in a prefecture, respectively. The proportion of wide-area epidemics in epidemic weeks was 62%. The average number of wide-area epidemic weeks for pediatric diseases varied among diseases; it was more than 4 weeks for infectious gastroenteritis and herpangina and less than 1 week for pertussis, rubella, and measles. The proportion of wide-area epidemics in epidemic weeks was 28-41% for infectious gastroenteritis, hand-foot-mouth disease, and herpangina and less than 20% for other diseases.

**CONCLUSIONS:**

The frequency of wide-area epidemics of influenza and pediatric diseases in various prefectures was observed. Epidemics of infectious diseases such as influenza and herpangina occurring in small areas were likely to spread to wide areas.

Epidemics of infectious diseases usually start in small areas and subsequently become widespread widely. To control and prevent epidemics in a wide area, such as a prefecture or nation, it is essential to observe epidemics in small areas, such as municipalities, and to determine whether these epidemics can occur over a wide area (a wide-area epidemic). Hence, infectious disease surveillance systems have been implemented in many countries,^1^^-^^6^ and several methods have been used for detecting epidemics. In Japan, a method for detecting epidemics in public health center (PHC) areas has previously been proposed,^7^^,^^8^ evaluated,^9^^,^^10^ and used in the National Epidemiological Surveillance of Infectious Diseases (NESID).^6^ No criteria have been established for wide-area epidemics, and these types of epidemics have not been fully investigated.

In the present study, we attempted to determine the occurrence of wide-area epidemics in various prefectures using the information on epidemics in PHC areas in the prefectures by the abovementioned method. Based on the NESID data of Japan obtained from 1999 through 2005, we found wide-area epidemics of influenza and pediatric diseases in these prefectures.

## METHODS

### Epidemiologic Surveillance of Infectious Diseases in Japan

The NESID in Japan is organized by the Ministry of Health, Labour and Welfare, and it is controlled by the Infectious Disease Surveillance Center, National Institute of Infectious Diseases, Japan.^5^^,^^6^ The NESID has targeted influenza and pediatric diseases for sentinel surveillance. According to the NESID guidelines, local governments (prefectures) select sentinel clinics and hospitals for influenza and pediatric diseases from pediatric and internal medicine departments.^11^ The number of sentinel clinics and hospitals in a PHC area is approximately proportional to the population size. The sentinel clinics and hospitals send information on the numbers of patients with targeted diseases to the PHCs on a weekly basis. These data are then used to monitor trends and variations in the number of cases of influenza and pediatric diseases, to detect epidemics in the PHC areas, and to estimate incidence rates in the entire country.^6^^,^^12^

### Surveillance Data and Method for Detecting Epidemics in PHC Areas

We used the data obtained from the NESID in Japan for fiscal years 1999-2005. Fiscal year 1999 in Japan means the period from April 1999 through March 2000. The numbers of cases of influenza and pediatric diseases per sentinel clinic and hospital reported weekly in the PHC area were used as indices for the analysis. The list of the diseases is shown in Table 1. Following the integration of PHCs in Japan, the number of centers has changed drastically. Table 2 shows the number of PHCs for fiscal years 1999-2003, 2004, and 2005, and distribution of PHC population size in each prefecture. In fiscal year 1999-2003, the number of PHCs was 568, which was the number of PHCs operational on April 1, 2003. Furthermore, the number of PHCs for fiscal years 2004 and 2005 was 547 and 545, respectively.

**Table 1.** Critical values for epidemics in public health center areas.

Disease	Critical value*	

	onset	end
Influenza	30	10
Pharyngoconjunctival fever	2	0.1
Group A streptococcal pharyngitis	4	2
Infectious gastroenteritis	20	12
Chickenpox	7	4
Hand-foot-mouth disease	5	2
Erythema infectiosum	2	1
Pertussis	1	0.1
Rubella	1	0.1
Herpangina	6	2
Measles	1.5	0.5
Mumps	6	2

* : Units indicate the number of cases in sentinel clinics and hospitals in an area over the course of a week.

**Table 2.** Population and number of public health centers in various prefectures.

Prefecture	Population	Population in the PHC area				Number of public health centers (Fiscal year)		
	
		Minimum	%	Maximum	%	1999-2003	2004	2005
Hokkaido	5,683,062	27,340	0.5	1,822,368	32.1	29	30	30

Aomori	1,475,728	87,366	5.9	355,214	24.1	6	6	6
Iwate	1,416,180	69,222	4.9	490,736	34.7	10	10	10
Miyagi	2,365,320	84,947	3.6	429,051	18.1	12	12	12
Akita	1,189,279	45,419	3.8	317,625	26.7	9	9	9
Yamagata	1,244,147	95,410	7.7	581,488	46.7	4	4	4
Fukushima	2,126,935	34,988	1.6	518,385	24.4	8	8	8

Ibaraki	2,985,676	75,793	2.5	493,888	16.5	12	12	12
Tochigi	2,004,817	207,899	10.4	473,435	23.6	6	6	6
Gunma	2,024,852	67,724	3.3	385,951	19.1	11	11	11
Saitama	6,938,006	117,777	1.7	1,269,216	18.3	22	22	22
Chiba	5,926,285	86,210	1.5	887,164	15.0	16	16	16
Tokyo	12,064,101	27,640	0.2	912,138	7.6	31	31	31
Kanagawa	8,489,974	52,253	0.6	605,561	7.1	38	38	38

Niigata	2,475,733	56,409	2.3	527,324	21.3	14	14	14
Toyama	1,120,851	134,411	12.0	325,700	29.1	5	5	5
Ishikawa	1,180,977	89,323	7.6	456,438	38.6	5	5	5
Fukui	828,944	63,546	7.7	278,755	33.6	6	6	6

Yamanashi	888,172	67,022	7.5	299,972	33.8	8	8	8
Nagano	2,215,168	42,159	1.9	424,883	19.2	11	11	11
Gifu	2,107,700	116,723	5.5	402,751	19.1	8	8	8
Shizuoka	3,767,393	52,431	1.4	763,855	20.3	11	11	9
Aichi	7,043,300	62,625	0.9	499,664	7.1	32	31	31
Mie	1,857,339	45,045	2.4	358,572	19.3	9	9	9

Shiga	1,342,832	55,451	4.1	309,793	23.1	7	7	7
Kyoto	2,644,391	11,917	0.5	290,538	11.0	23	18	18
Osaka	8,805,081	250,806	2.8	2,598,774	29.5	17	18	18
Hyogo	5,550,574	22,337	0.4	1,493,398	26.9	29	17	17
Nara	1,442,795	45,565	3.2	452,652	31.4	6	6	6
Wakayama	1,069,912	44,015	4.1	386,551	36.1	9	8	8

Tottori	613,289	116,686	19.0	249,385	40.7	3	3	3
Shimane	761,503	25,239	3.3	256,819	33.7	7	7	7
Okayama	1,950,828	38,492	2.0	626,642	32.1	10	11	11
Hiroshima	2,878,915	56,870	2.0	1,126,239	39.1	10	10	10
Yamaguchi	1,527,964	43,473	2.8	289,829	19.0	10	9	9

Tokushima	824,108	27,166	3.3	448,770	54.5	6	6	6
Kagawa	1,022,890	36,014	3.5	425,996	41.6	5	4	4
Ehime	1,493,092	69,713	4.7	473,379	31.7	9	7	7
Kochi	813,949	62,566	7.7	330,654	40.6	6	6	6

Fukuoka	5,015,699	93,581	1.9	1,011,471	20.2	22	22	22
Saga	876,654	81,457	9.3	362,090	41.3	5	5	5
Nagasaki	1,516,523	33,538	2.2	423,167	27.9	10	10	10
Kumamoto	1,859,344	59,261	3.2	662,012	35.6	11	11	11
Oita	1,221,140	28,689	2.3	436,470	35.7	10	10	10
Miyazaki	1,170,007	26,367	2.3	305,755	26.1	9	9	9
Kagoshima	1,786,194	13,875	0.8	552,098	30.9	15	14	14

Okinawa	1,318,220	48,705	3.7	446,403	33.9	6	6	6

Total	126,925,843					568	547	545

The population size is calculated from the year 2000 census.

In the population in the PHC area, each number shows the minimum/maximum PHC population size in a given prefecture.

The percentage of minimum/maximum PHC shows proportion of population size in a given prefecture.

The method for detecting epidemics in the PHC areas in the NESID has been described previously.^8^^,^^9^ A brief description of this method is as follows. The method is based on an index calculated from the number of cases per sentinel clinic and hospital in a PHC area over a week. An epidemic in a PHC area was considered to occur when the index in the area exceeded the critical value for epidemic onset and continued until the index in that area was lower than the critical value for the end of the epidemic. Table 1 shows the critical values for the onset and end of epidemics of various diseases. The critical values were determined according to the distribution of the number of cases per week per sentinel clinic and hospital using the surveillance data.^8^^,^^10^

### Method for Detecting Epidemics in Prefectures and Method of Analysis

When an epidemic occurred in at least one PHC area in a given prefecture, the prefecture was considered to have an epidemic. The proportion of people living in PHC areas with epidemics in a prefecture was selected as the index for wide-area epidemics. When this index exceeded 30% of the prefectural population, the prefecture was considered to have a wide-area epidemic. These epidemics were considered to have ended if the index decreased to below 30%. The population size of each PHC area and prefecture was calculated from the year 2000 census in Japan (Table 2).

**Figure 1.**
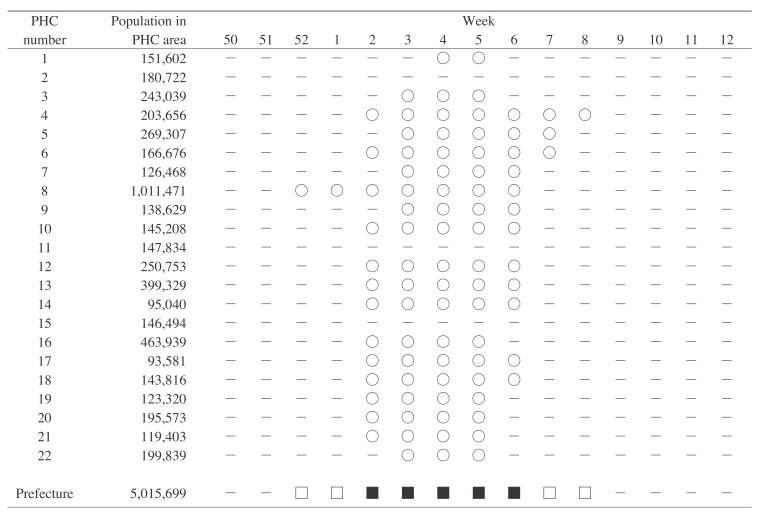
Influenza epidemics in a specific prefecture in the 2005/2006 season. The figure shows the occurrence of epidemics in one prefecture and its public health center (PHC) areas from week 50 of 2005 through week 12 of 2006. Each PHC number shows the PHC area in the prefecture, and the symbol in each week represents the presence or absence of an epidemic. ○ Epidemic in a PHC area. – No epidemic in a PHC area or prefecture. □ Epidemic in the prefecture. ■ Wide-area epidemic in the prefecture.

Figure 1 shows influenza epidemics in a specific prefecture. In this prefecture, epidemics started occurring in each PHC at the beginning of the year. Because there were many epidemics in the PHC areas in week 2 of 2006, a wide-area epidemic commenced. After 4 weeks, the number of epidemics in the PHC areas decreased, and the wide-area epidemic was terminated by the end of week 6. Thus, in this prefecture, wide-area epidemics occurred for 5 weeks, while epidemics occurred for 9 weeks from week 52 of 2005 through week 8 of 2006.

With regard to influenza and pediatric diseases, we determined the number of weeks for which epidemics/wide-area epidemics occurred in prefectures in fiscal years 1999-2005. We also determined the average number of weeks for which epidemics/wide-area epidemics occurred and the proportion of wide-area epidemics (the number of weeks for which wide-area epidemics occurred divided by those for which epidemics occurred and multiplied by 100).

## RESULTS

### Epidemics of Influenza in Prefectures

**Figure 2.**
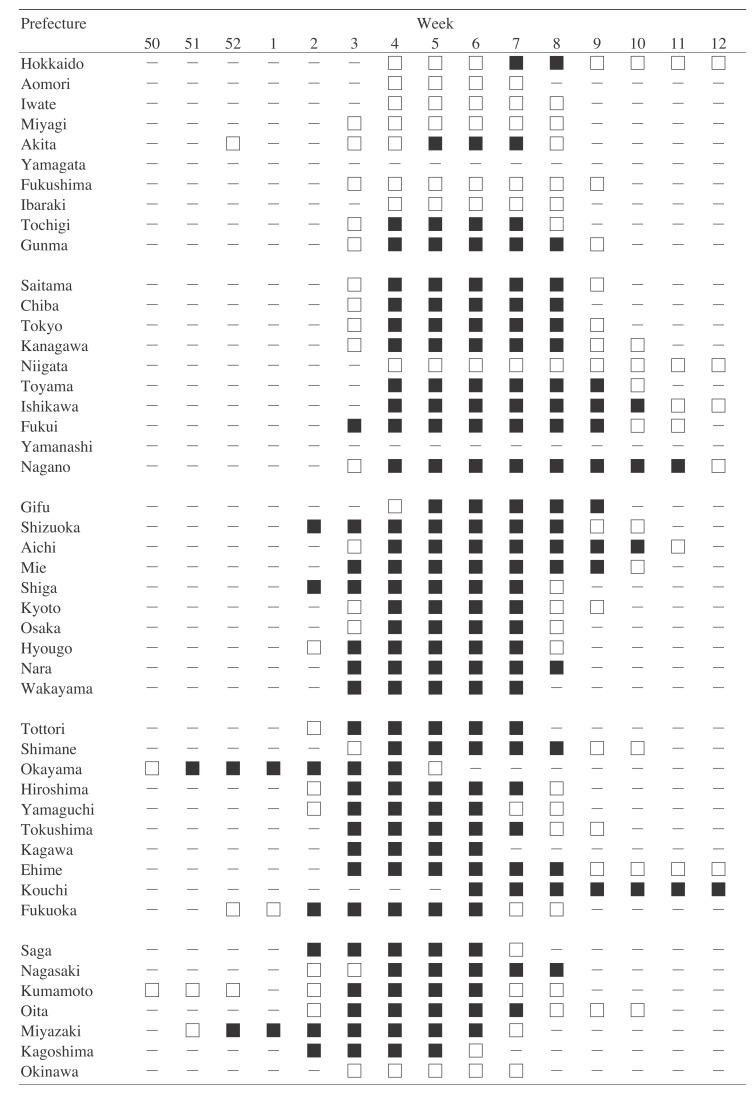
Influenza epidemics in various prefectures in the 2005/2006 season. The figure shows the occurrence of epidemics and wide-area epidemics in various prefectures from week 50 of 2005 through week 12 of 2006. – No epidemic. □ Epidemic. ■ Wide-area epidemic.

Figure 2 shows the influenza epidemics in 47 prefectures in the 2005/2006 season. The prefectures are listed in order from the northern to the southern/western prefectures of Japan. In some northern prefectures, epidemics occurred for several weeks; however, wide-area epidemics did not. In many other prefectures, wide-area epidemics occurred for 4 or 5 weeks, while epidemics occurred for around 7 weeks. Table 3 shows the number of epidemic weeks/wide-area

**Table 3.** Annual number of epidemic weeks for influenza in various prefectures in fiscal years 1999-2005.

Prefecture	Fiscal year							

	1999	2000	2001	2002	2003	2004	2005	1999-2005
Hokkaido	3 / 11	0/ 2	0 / 14	3 / 19	3 / 12	7/ 8	2 / 13	18 / 79

Aomori	5/ 6	0/ 2	0/ 9	5/ 8	3 / 10	6/ 6	0/ 6	19 / 47
Iwate	4/ 8	0/ 4	5 / 10	8/ 9	5/ 6	6/ 8	0 / 10	28 / 55
Miyagi	5 / 13	0/ 3	4/ 7	6 / 10	4/ 6	6 / 11	0/ 6	25 / 56
Akita	6/ 7	2/ 4	4/ 7	9 / 10	6/ 9	5/ 7	3 / 12	35 / 56
Yamagata	5/ 7	0/ 0	0/ 7	8 / 10	0/ 7	7/ 8	0/ 0	20 / 39
Fukushima	4/ 6	0/ 3	4/ 7	8/ 8	6/ 7	7/ 9	0/ 7	29 / 47

Ibaraki	0/ 4	0/ 0	0/ 0	4/ 9	3/ 5	7/ 8	0/ 5	14 / 31
Tochigi	4/ 6	0/ 0	5/ 8	7/ 8	3/ 4	8/ 8	4/ 6	31 / 40
Gunma	4/ 6	0/ 0	7 / 10	5 / 10	4/ 8	7/ 9	5/ 7	32 / 50
Saitama	5/ 9	0/ 2	5/ 8	7/ 8	5/ 6	8/ 9	5/ 7	35 / 49
Chiba	4/ 7	0/ 2	3/ 8	7 / 11	5/ 5	7/ 8	5/ 6	31 / 47
Tokyo	3/ 6	0/ 0	0/ 6	5/ 8	3/ 5	7/ 9	5/ 7	23 / 41
Kanagawa	4/ 8	0/ 0	2/ 8	6 / 10	5/ 5	6 / 10	5/ 8	28 / 49

Niigata	6/ 9	0/ 3	5/ 6	9 / 12	6/ 8	8 / 10	0 / 12	34 / 60
Toyama	6/ 9	0/ 0	0/ 6	8/ 9	5/ 5	7/ 9	6/ 7	32 / 45
Ishikawa	7/ 7	0/ 2	4/ 6	11 / 12	5/ 7	7/ 8	7/ 9	41 / 51
Fukui	5/ 6	0/ 0	0/ 3	12 / 12	8/ 8	7/ 7	7 / 13	39 / 49

Yamanashi	5/ 6	0/ 3	0/ 5	8/ 8	4/ 6	5/ 7	0/ 0	22 / 35
Nagano	6/ 7	0/ 0	1/ 6	8 / 13	6/ 7	8/ 8	8 / 10	37 / 51
Gifu	4/ 7	0/ 0	3/ 4	5/ 6	4/ 5	7/ 9	5/ 6	28 / 37
Shizuoka	6/ 7	0/ 4	6/ 7	8 / 10	5/ 7	8 / 10	7/ 9	40 / 54
Aichi	4 / 11	0/ 0	7/ 9	7/ 9	5 / 11	8/ 9	7 / 13	38 / 62
Mie	5/ 9	0/ 4	4 / 10	6 / 11	5/ 6	9 / 10	7 / 12	36 / 62

Shiga	4/ 4	0/ 0	0/ 0	8/ 9	5/ 6	6/ 7	6/ 7	29 / 33
Kyoto	4/ 6	0/ 0	0/ 4	5 / 13	3/ 8	6/ 8	4/ 7	22 / 46
Osaka	0/ 4	0/ 4	0/ 0	0/ 9	4/ 5	6/ 8	4/ 6	14 / 36
Hyogo	4 / 10	0/ 6	0 / 10	5 / 15	4/ 9	7/ 8	5/ 7	25 / 65
Nara	3/ 8	0/ 3	0/ 0	7/ 9	4/ 5	5/ 7	6/ 6	25 / 38
Wakayama	4/ 6	0/ 5	0/ 0	9 / 12	4/ 7	6/ 8	5/ 5	28 / 43

Tottori	5/ 6	0/ 0	8/ 8	10 / 11	5/ 7	7/ 7	5/ 6	40 / 45
Shimane	5/ 7	0/ 0	0/ 0	5 / 12	0/ 5	6/ 8	5 / 13	21 / 45
Okayama	5/ 6	0/ 0	0/ 0	9 / 11	5/ 6	6/ 8	6/ 8	31 / 39
Hiroshima	4/ 5	0/ 0	4/ 5	5/ 8	5/ 5	6/ 7	5/ 7	29 / 37
Yamaguchi	6/ 8	0/ 1	0 / 11	12 / 13	4 / 10	8/ 9	4/ 7	34 / 59

Tokushima	4/ 8	3/ 7	0/ 5	10 / 11	5/ 6	6/ 7	5/ 7	33 / 51
Kagawa	0/ 3	0/ 0	0/ 0	10 / 10	0/ 0	6/ 6	4/ 4	20 / 23
Ehime	6/ 7	2/ 3	4/ 9	6 / 11	5/ 7	6/ 6	6 / 10	35 / 53
Kochi	5/ 8	3/ 6	0/ 8	10 / 11	0/ 4	7/ 8	7/ 7	32 / 52

Fukuoka	6 / 10	0/ 0	0 / 10	14 / 17	5/ 8	8/ 9	5/ 9	38 / 63
Saga	4/ 6	0/ 2	0/ 0	14 / 17	0/ 5	9/ 9	5/ 6	32 / 45
Nagasaki	5/ 7	0/ 4	7 / 11	9 / 13	7 / 10	8/ 9	5/ 8	41 / 62
Kumamoto	4/ 7	0/ 0	4/ 9	11 / 16	3/ 7	7/ 9	4 / 10	33 / 58
Oita	7/ 8	0/ 0	10 / 11	12 / 16	6/ 9	6/ 9	5 / 10	46 / 63
Miyazaki	6/ 7	0/ 2	0 / 12	8 / 12	7/ 9	9/ 9	7 / 10	37 / 61
Kagoshima	5/ 6	0/ 4	4/ 6	12 / 12	7/ 7	8 / 10	4/ 5	40 / 50

Okinawa	4/ 5	0/ 0	0/ 0	12 / 12	5/ 7	6/ 7	0/ 9	27 / 40

Total	210/ 334	10 / 85	110 / 290	373 / 520	201/ 317	323/ 388	200 / 365	1,427/ 2,299

Mean	4.5 / 7.1	0.2 / 1.8	2.3 / 6.2	7.9 / 11.1	4.3 / 6.7	6.9 / 8.3	4.3 / 7.8	4.3 / 7.0

Proportion	62.9	11.8	37.9	71.7	63.4	83.2	54.8	62.1

PHC: Public health center

The columns show the total number of wide-area epidemics/epidemics in various prefectures for each year and for the total observation period. Mean is the average number of wide-area epidemics/epidemics. Proportion is the percentage of wide-area epidemic weeks divided by epidemic weeks.

epidemic weeks for influenza in 47 prefectures in fiscal years 1999-2005. Many epidemics occurred in 1999, 2002, 2003, 2004, and 2005, with the average number of weeks of epidemics ranging from 6.7 to 11.1. During these years, many wide-area epidemics were also observed, and the average number of weeks of wide-area epidemics ranged from 4.3 to 7.9, with a proportion of more than 50%. In contrast, both epidemics and wide-area epidemics were not observed to occur with considerable frequency in 2000 and 2001. The average number of epidemic weeks was 6.2 and below. In these 2 years, the average number of weeks of wide-area epidemics was below 2.3, and the proportion of epidemics decreased to below 50%. When the proportion of epidemics in the prefectures over the 7-year period was compared, a variability ranging from 20% to almost 90% was found. A total of 2299 and 1427 weeks of epidemics and wide-area epidemics, respectively, were recorded. On average, 7.0 epidemic weeks and 4.3 wide-area epidemic weeks were observed in the prefectures during the total observation period. The proportion of wide-area epidemics in epidemic weeks was 62.1%.

### Epidemics of Pediatric Diseases in Prefectures

Table 4 shows the variability in the annual number of weeks for epidemics and wide-area epidemics of pediatric diseases in fiscal years 1999-2005. The number of epidemic weeks, the number of wide-area epidemic weeks, and the proportion of wide-area epidemics are shown. The average number of epidemic weeks in a year in a prefecture was 1.1-5.4 weeks for pertussis, rubella, and measles and more than 10 weeks for other diseases.

**Table 4.** Annual number of epidemic weeks for pediatric diseases in fiscal years 1999-2005.

Disease	Fiscal year								

	1999	2000	2001	2002	2003	2004	2005	1999-2005	
Pharyngoconjunctival fever									
Wide-area epidemic weeks	7	81	78	29	204	352	200	951	(2.9)
Epidemic weeks	240	543	558	424	1,107	1,300	1,216	5,388	(16.4)
Proportion (%)	2.9	14.9	14.0	6.8	18.4	27.1	16.4	17.7	

Group A streptococcal pharyngitis									
Wide-area epidemic weeks	58	138	140	100	203	188	230	1,057	(3.2)
Epidemic weeks	825	1,193	979	880	1,095	1,282	1,063	7,317	(22.2)
Proportion (%)	7.0	11.6	14.3	11.4	18.5	14.7	21.6	14.4	

Infectious gastroenteritis									
Wide-area epidemic weeks	224	196	152	156	204	212	223	1,367	(4.2)
Epidemic weeks	691	801	684	652	679	694	635	4,836	(14.7)
Proportion (%)	32.4	24.5	22.2	23.9	30.0	30.5	35.1	28.3	

Chickenpox									
Wide-area epidemic weeks	59	66	30	31	27	46	16	275	(0.8)
Epidemic weeks	547	720	491	523	536	376	425	3,618	(11.0)
Proportion (%)	10.8	9.2	6.1	5.9	5.0	12.2	3.8	7.6	

Hand-foot-mouth disease									
Wide-area epidemic weeks	40	346	156	56	268	75	77	1,018	(3.1)
Epidemic weeks	240	855	522	407	696	435	348	3,503	(10.6)
Proportion (%)	16.7	40.5	29.9	13.8	38.5	17.2	22.1	29.1	

Erythema infectiosum									
Wide-area epidemic weeks	25	33	151	96	13	53	64	435	(1.3)
Epidemic weeks	373	538	906	675	402	479	414	3,787	(11.5)
Proportion (%)	6.7	6.1	16.7	14.2	3.2	11.1	15.5	11.5	

Pertussis									
Wide-area epidemic weeks	0	0	0	0	0	0	0	0	(0.0)
Epidemic weeks	87	104	36	40	31	37	11	346	(1.1)
Proportion (%)	0.0	0.0	0.0	0.0	0.0	0.0	0.0	0.0	

Rubella									
Wide-area epidemic weeks	2	0	0	8	15	5	0	30	(0.1)
Epidemic weeks	137	73	67	86	136	138	2	639	(1.9)
Proportion (%)	1.5	0.0	0.0	9.3	11.0	3.6	0.0	4.7	

Herpangina									
Wide-area epidemic weeks	284	186	220	122	261	140	199	1,412	(4.3)
Epidemic weeks	616	492	460	404	553	434	514	3,473	(10.6)
Proportion (%)	46.1	37.8	47.8	30.2	47.2	32.3	38.7	40.7	

Measles									
Wide-area epidemic weeks	47	117	116	16	15	0	0	311	(0.9)
Epidemic weeks	246	601	569	236	101	11	0	1,764	(5.4)
Proportion (%)	19.1	19.5	20.4	6.8	14.9	0.0	0.0	17.6	

Mumps									
Wide-area epidemic weeks	20	84	260	75	0	32	133	604	(1.8)
Epidemic weeks	276	675	1,278	722	211	482	763	4,407	(13.4)
Proportion (%)	7.2	12.4	20.3	10.4	0.0	6.6	17.4	13.7	

PHC : Public health center

The columns show the total number of weeks for which a wide-area epidemic or an epidemic occurred each year.

The proportion is the percentage of wide-area epidemic weeks divided by epidemic weeks. The number of weeks per year

per prefecture is given in parentheses.

The average number of wide-area epidemic weeks ranged from several weeks to very few. It was less than 1 week for chickenpox, pertussis, rubella, and measles; 1.0-2.9 weeks for pharyngoconjunctival fever, erythema infectiosum, and mumps; and 3.0-4.3 weeks for group A streptococcal pharyngitis, infectious gastroenteritis, hand-foot-mouth disease, and herpangina. The trends in the number of wide-area epidemic weeks varied among diseases with increasing trends for pharyngoconjunctival fever and group A streptococcal pharyngitis; a decreasing trend for measles; and fluctuations for hand-foot-mouth disease, erythema infectiosum, and mumps. The proportion of wide-area epidemics in epidemic weeks was 40.7% for herpangina, 28.3-29.1% for infectious gastroenteritis and hand-foot-mouth disease, and less than 20.0% for other diseases.

## DISCUSSION

Using the infectious disease surveillance data of 1999-2005, we investigated epidemics and wide-area epidemics of influenza and pediatric diseases in various prefectures in Japan. Epidemics and wide-area epidemics of influenza occurred for an average of 7.0 and 4.3 weeks, respectively in a year in a given prefecture. The occurrence of wide-area epidemics in prefectures was not expected to be frequent when compared with epidemics in PHC areas.^9^^,^^13^ The proportion of wide-area epidemics in epidemic weeks was 62%. This implied that when the number of influenza cases increased and an epidemic started in a certain PHC area, the disease was likely to spread over a prefecture.^1^^,^^3^

Few epidemics of pertussis, rubella, and measles were observed during the 7-year period. Hence, very few wide-area epidemics occurred. These results were mainly attributed to the vaccination program against pertussis, rubella, and measles in Japan.^14^^-^^16^ Wide-area epidemics of other diseases were observed among many epidemics. The proportion of wide-area epidemics in epidemic weeks was 41% for herpangina, suggesting that the epidemic in small areas was likely to spread over wide areas, similar to influenza. With many other diseases, the proportion of wide-area epidemics was less than 20%. These findings would be useful for public health practices against these diseases in the prefectures.

A previously reported method for detecting epidemics in small areas was used in this analysis. This method has been used as a part of the epidemic alert system in the NESID in Japan.^6^^,^^9^ It would be reasonable to assume that most wide-area epidemics of infectious diseases start from aberrations of cases in small PHC areas. Based on this rationale, epidemic information for PHC areas was used in our analysis during the detection of epidemics or wide-area epidemics in a prefecture.

The method for early detection of epidemics in PHC areas has been established and is in operation in the infectious surveillance system in Japan.^6^^,^^9^ Thus, although there are several approaches to define a wide-area epidemic based on infectious disease surveillance data, it is practical to utilize this resource for the detection of wide-area epidemics.

Our study has several limitations. A wide-area epidemic was defined to occur when the proportion of people living in PHC areas with epidemics in a prefecture exceeded 30% of the prefectural population. The number of PHCs in a prefecture ranged from 3 to 30. In some prefectures with small number of PHCs, only one PHC dominated more than 30 % population in a prefecture (Table 2). If the number of PHCs was small and/or one PHC dominated over half population in a prefecture, an epidemic in only one PHC would greatly affect the issue of wide-area epidemics. When we interpret a wide-area epidemic in a given prefecture, we must check the PHC distribution in a prefecture. The criterion for a wide-area epidemic was fixed at 30% for all diseases. Although this criterion worked well for influenza and some other pediatric diseases, as there were a fair number of wide-area epidemics of influenza that occurred each year, a more apt criterion may be needed for improvement for those diseases with relatively few cases. In our definition of a wide-area epidemic, the prefecture is the unit of a wide-area epidemic. When an epidemic occurred in-between prefectures, we can not detect this epidemic from the proposed method.

As a countermeasure against epidemics of infectious diseases, an alert for wide-area epidemics is an important issue in public health practice. Although some difficulties exist with respect to the alert issue, such as the purpose for an alert, the definition of an epidemic, and countermeasures for control, the development of an alert system is necessary.^7^^,^^8^^,^^10^ We believe that this study will help to promote further discussion on this important issue.
